# P-37. Factors Associated with Healthcare Professionals’ RSV Older Adult Vaccination Knowledge and Practices During the First Season of Vaccine Availability in the United States

**DOI:** 10.1093/ofid/ofae631.244

**Published:** 2025-01-29

**Authors:** Elizabeth M La, Carolyn Sweeney, David Singer, Eric Davenport, Sarah Calhoun, Andrea Harmelink

**Affiliations:** GSK, Philadelphia, Pennsylvania; RTI-HS, Research Triangle Park, North Carolina; GSK, Philadelphia, Pennsylvania; RTI Health Solutions, Triangle Research Park, North Carolina; RTI Health Solutions, Triangle Research Park, North Carolina; GSK, Philadelphia, Pennsylvania

## Abstract

**Background:**

In 2023, respiratory syncytial virus (RSV) vaccines were approved by the United States (US) Food and Drug Administration (FDA) for adults aged ≥60 years and recommended by the Centers for Disease Control and Prevention’s Advisory Committee on Immunization Practices (ACIP) using shared clinical decision-making (SCDM). This study evaluated healthcare professional (HCP) characteristics associated with RSV vaccination knowledge and practices.

**Figure:**
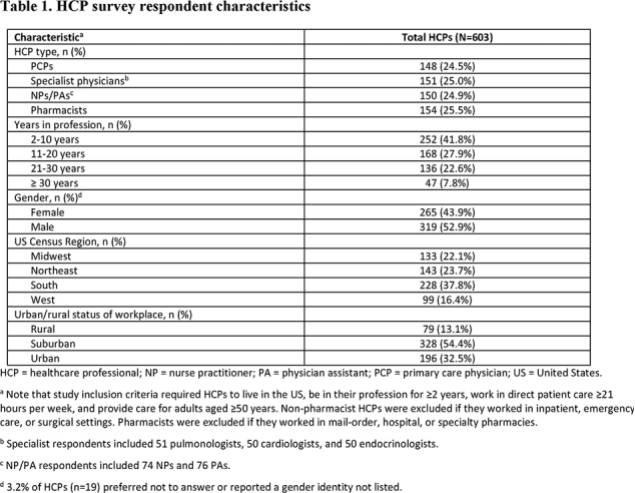

**Methods:**

A cross-sectional online survey targeting approximately 600 HCPs was conducted in November 2023 to assess HCP knowledge, attitudes, and practices related to older adult RSV vaccination. Three logistic regression models used these survey data to explore HCP characteristics associated with RSV vaccination knowledge and practices (e.g., likelihood of knowing FDA indication and ACIP recommendation). The models included HCP type as a covariate a priori, with other covariates determined using backwards selection with a prespecified stay criterion (*P* < 0.1).

**Figure:**
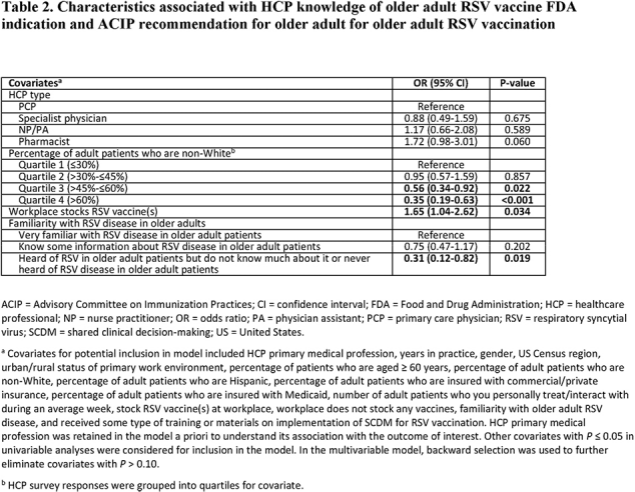

**Results:**

The survey was completed by 603 HCPs (Table 1). HCPs had higher odds of being knowledgeable about the FDA indication and ACIP recommendation for older adult RSV vaccination if their workplace stocked RSV vaccine(s); odds were lower if HCPs were least familiar with RSV disease or had a higher percentage of non-White adult patients (Table 2). Odds of initiating RSV vaccination conversations with ≤5% of older adult patients were higher among HCPs who were less familiar with RSV disease, felt less confident in SCDM conversations, and perceived RSV protection to be less important than other respiratory infections (Table 3). Being less familiar with RSV disease was also associated with higher odds of not recommending/administering RSV vaccines to any older adults in the past 3 months (Table 4).

**Figure:**
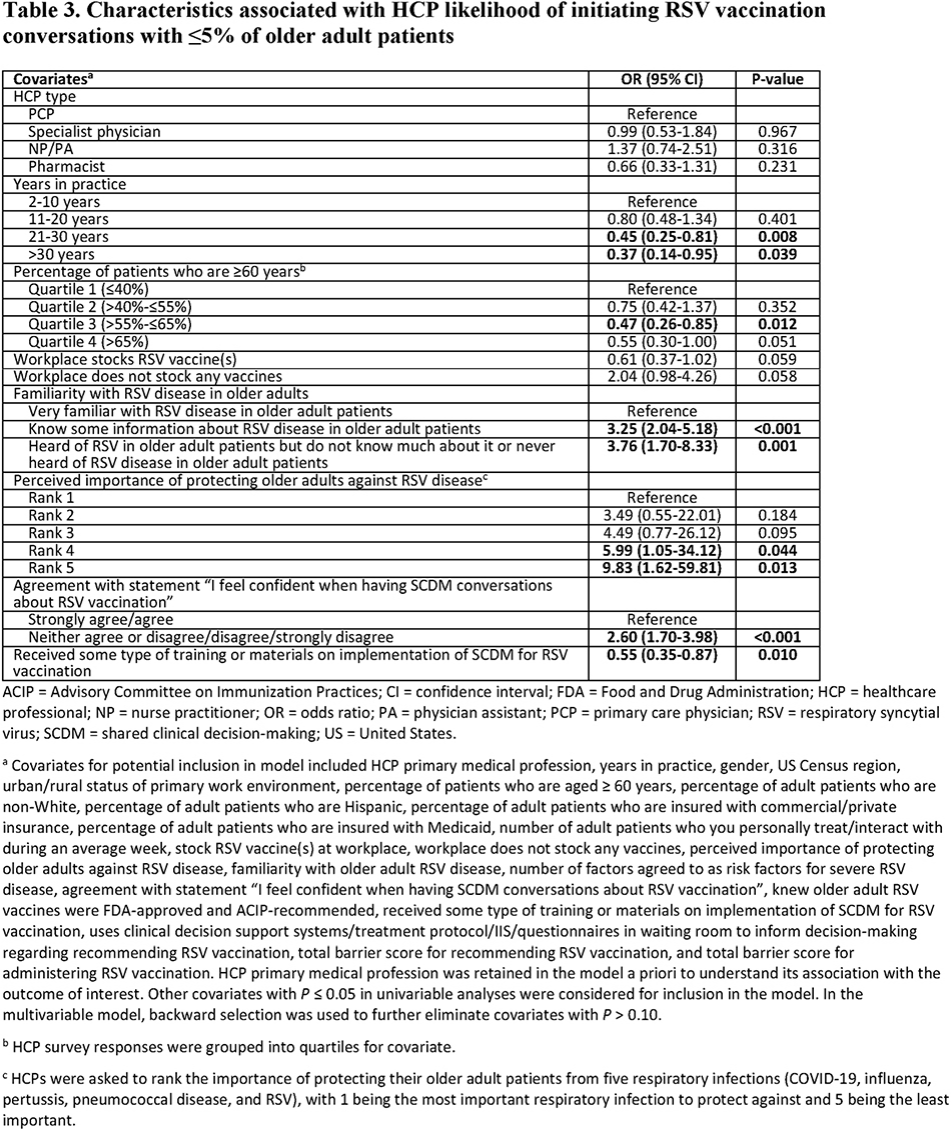

**Conclusion:**

HCP familiarity with older adult RSV disease was associated with RSV vaccination knowledge and practices, highlighting the importance of continued RSV disease awareness efforts. Results can be used to tailor these efforts towards HCPs with potential vaccination knowledge and practice gaps to help ensure equitable RSV vaccination access across older adults.

**FUNDING:** GSK (study identifier: VEO-000726)

**Figure:**
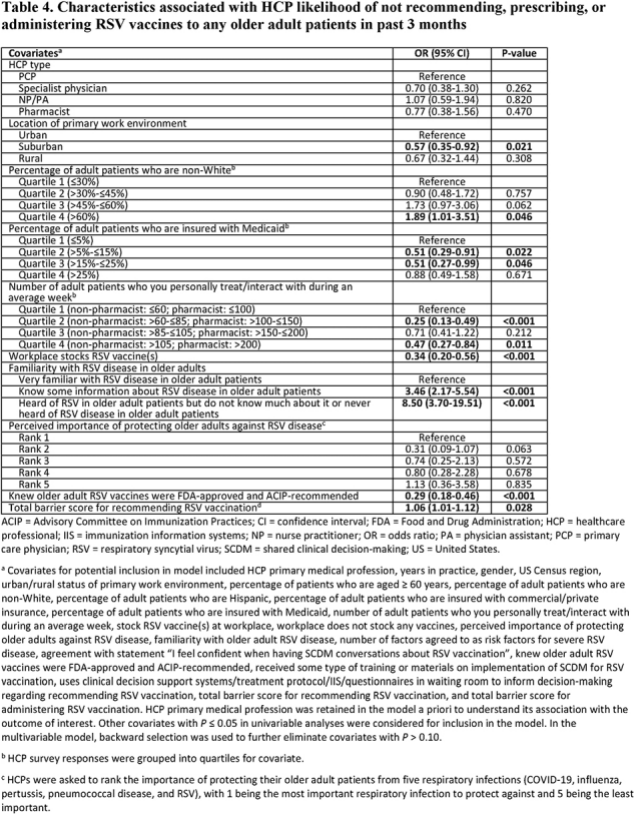

**Disclosures:**

**Elizabeth M. La, PhD**, GSK: employee|GSK: Stocks/Bonds (Private Company) **David Singer, PharmD, MS**, GSK: employee|GSK: Stocks/Bonds (Public Company) **Eric Davenport, MStat, MEcon**, GSK: study funding **Andrea Harmelink, PhD, FNP**, GSK: Stocks/Bonds (Public Company)

